# Recessive Loss of *PI4K2A* Function Causes a Developmental and Epileptic Dyskinetic Encephalopathy with Prominent Orolingual Dyskinesia

**DOI:** 10.1002/mds.30286

**Published:** 2025-08-07

**Authors:** Reza Maroofian, Juan Darío Ortigoza‐Escobar, Pooja Rohilla, Javeria Raza Alvi, Aziza M. Mushiba, Naif A.M. Almontashiri, Stephanie Efthymiou, Tipu Sultan, Tamas Balla, Henry Houlden

**Affiliations:** ^1^ Department of Neuromuscular Disorders UCL Queen Square Institute of Neurology London United Kingdom; ^2^ Movement Disorders Unit, Pediatric Neurology Department Institut de Recerca Hospital Sant Joan de Déu Barcelona Barcelona Spain; ^3^ U‐703 Centre for Biomedical Research on Rare Diseases (CIBER‐ER) Instituto de Salud Carlos III Barcelona Spain; ^4^ European Reference Network for Rare Neurological Diseases (ERN‐RND) Barcelona Spain; ^5^ Section on Molecular Signal Transduction Eunice Kennedy Shriver, National Institute of Child Health and Human Development, National Institutes of Health Bethesda Maryland USA; ^6^ Department of Pediatric Neurology Institute of Child Health, Children Hospital Lahore Lahore Pakistan; ^7^ Clinical Genetic Section, Pediatric Subspecialties Department Children's Specialized Hospital, King Fahad Medical City Riyadh Saudi Arabia; ^8^ Center for Genetics and Inherited Diseases and Clinical Laboratory Sciences, Taibah University Madinah Saudi Arabia; ^9^ Research Department King Khaled Eye Specialist Hospital Riyadh Saudi Arabia

**Keywords:** Orolingual dyskinesia, PI4K2A, neurodevelopmental disorders, genetic variants, pediatric movement disorders, developmental and epileptic encephalopathies

## Abstract

**Background:**

Biallelic loss‐of‐function variants in *PI4K2A* have been associated with a neurodevelopmental disorder characterized by seizures and movement disorders, including orofacial dyskinesia. However, only 4 cases have been reported. Orolingual dyskinesia—defined as involuntary movements of the mouth and tongue—is observed in various pediatric neurodevelopmental disorders (NDD) but remains under‐recognized.

**Objectives:**

The aims were to highlight orolingual dyskinesia as a core feature of *PI4K2A*‐related disorder (*PI4K2A*‐RD) and explore its presence across other NDDs.

**Methods:**

We described two new families with *PI4K2A*‐RD and reviewed the clinical features of four previously reported cases. A focused literature search was also conducted to identify other neurogenetic conditions associated with orolingual dyskinesia.

**Results:**

All individuals with *PI4K2A* deficiency exhibited orolingual dyskinesia, along with developmental delay, movement abnormalities, and variable seizures. The literature review confirmed frequent underreporting of this feature in NDDs.

**Conclusions:**

Orolingual dyskinesia is a relevant but under‐recognized clinical sign in *PI4K2A*‐RD and other neurogenetic conditions, with potential diagnostic value. © 2025 The Author(s). *Movement Disorders* published by Wiley Periodicals LLC on behalf of International Parkinson and Movement Disorder Society.

*PI4K2A* encodes phosphatidylinositol 4‐kinase type 2 α, a critical enzyme involved in phosphoinositide signaling and intracellular vesicular trafficking.^1^ Biallelic loss‐of‐function variants in *PI4K2A* cause a rare neurodevelopmental disorder (NDD) (*PI4K2A*‐related disorder [*PI4K2A*‐RD]), previously reported in only 4 patients, characterized by global developmental delay, epilepsy, failure to thrive, and dysmorphic features.^2^, ^3^ These cases suggest a developmental and epileptic dyskinetic encephalopathy (DEDE), a subset of genetic disorders marked by severe neurological impairment. However, the full phenotypic spectrum and molecular mechanisms of *PI4K2A*‐RD remain underexplored due to its rarity.

Here, we report two new patients with biallelic *PI4K2A* variants, expanding the cohort to six and providing original clinical and functional data. Genetic analysis identified novel homozygous variants, prompting detailed phenotyping and cellular studies to assess pathogenicity. A striking observation emerged: orolingual dyskinesia—involuntary movements of the mouth and tongue—was a consistent feature among living patients. This prompted us to investigate its role in *PI4K2A*‐RD and beyond.

Orolingual dyskinesia,^4^, ^5^, ^6^ although documented in various pediatric genetic^7^, ^8^, ^9^, ^10^, ^11^, ^12^ and acquired disorders,^13^, ^14^, ^15^ is often under‐recognized in DEDEs and NDDs. In children, it differs from adult‐onset causes (eg, neurodegenerative or drug induced) and may signal underlying trafficking or neuronal homeostasis defects. This study aims to define *PI4K2A*‐RD phenotype, validate its molecular basis, and explore broader diagnostic relevance of orolingual dyskinesia, leveraging this ultrarare condition to inform wider clinical practice.

## Patients and Methods

We studied two unreported patients with *PI4K2A*‐RD from consanguineous families (Pakistan and Saudi Arabia). Patients underwent neurological exams, developmental assessments, and video analysis by movement disorder specialists following standardized criteria.^4^ Brain magnetic resonance imaging (MRI) was available for patient 2 and an older sibling of patient 1. Videos from patient 2 and two prior cases^2^ characterized orolingual dyskinesia. Clinical and research exome sequencing was performed as described.^16^ Data of prior cases^2^, ^3^ were reviewed. Written informed consent was obtained from all new participants, and the study adhered to the guidelines of the Helsinki Declaration and the ethical committees of the involved institutions.

To assess variant impact, Green fluorescent protein (GFP)‐ and Near‐infrared fluorescent protein (iRFP)‐fused wild‐type and mutant PI4K2A (C1243T, C989_990del) proteins were expressed in *PI4K2A* knockout HEK293‐AT1 cells.^17^ Subcellular localization was examined using confocal microscopy, and phosphatidylinositol 4‐phosphate (PI4P) levels in Rab7‐positive endosomes were measured via bioluminescence resonance energy transfer (BRET) analysis (see Supporting Information for details).

A literature review of orofacial dyskinesias in genetic disorders was conducted (Supporting Information).

## Results

### Clinical Manifestations and Patient Characteristics

We identified two new patients with biallelic *PI4K2A* variants, expanding the cohort with four prior cases.^2^, ^3^

**FIG. 1:**
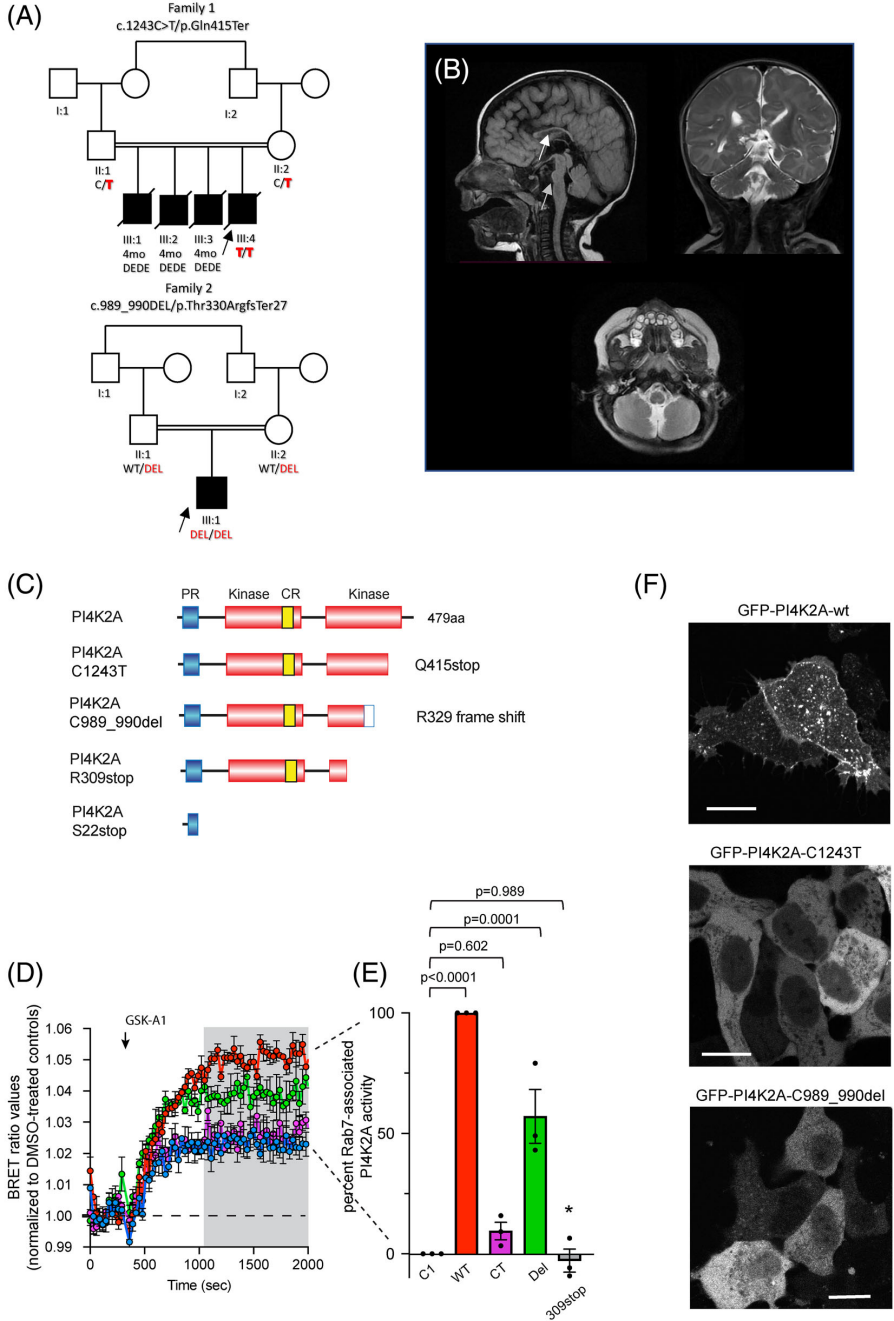
(**A**) Pedigrees and segregation results. Individuals with *PI4K2A*‐related disorders are indicated by filled black shapes with an arrow pointing to the proband. Open shapes represent unaffected individuals. Diagonal lines indicate deceased individuals. Squares represent males, and circles represent females. The presence of consanguinity between two individuals is represented by double lines. Below each individual are the generation number and the number of that individual in that generation. Segregation results for all individuals tested are indicated with either red (presence of the *PI4K2A* variant) or black (presence of the reference allele). Red/red text indicates the presence of *PI4K2A* variants in a homozygous state, whereas red/black text indicates the presence of the *PI4K2A* variant in a heterozygous state. III:1, III:2, and III:3 are male individuals who died at 4 months of age, presenting a DEDE (developmental and epileptic dyskinetic encephalopathy) phenotype similar to that of the index case III:4. (**B**) Brain MRI (magnetic resonance imaging) of P2 (sagittal, axial, and coronal views) reveals microcephaly, diffuse corpus callosum thinning suggestive of hypoplasia (white arrow), and a small hypoplastic pons (gray arrow). The midbrain, medulla, cerebellum, and vermis appear normal. Characterization of *PI4K2A* mutants using intact HEK293 cells. (**C**) Image of the PI4K2A sequence along with mutants relevant to this study. The proline‐rich (PR, blue) and kinase (orange) domains, along with the palmitoylated cysteine‐rich CCPCC motif (CR, yellow), are specifically highlighted. (**D**) Kinetics of PI4P (phosphatidylinositol 4‐phosphate) levels within the Rab7‐positive endosomal compartments of PI4K2A K/O cells expressing iRFP‐PI4K2A variants (wild type, or the indicated mutants) or a vector control (iRFP‐C1) in response to treatment with GSK‐A1 (30 nM). BRET (bioluminescence resonance energy transfer) measurements were performed using the sLuc‐P4M2x‐T2A‐mVenus‐Rab7 biosensor and are presented as mean values ± SEM (standard error of the mean) from three independent experiments carried out using triplicate wells. (**E**) Calculation of percentage rescue based on area under the curve (AUC) calculations using the BRET values for the time period labeled by the gray box. Recoveries were then calculated using the wild‐type enzyme values (red traces) as 100% and the values with the iRFP‐C1 (blue traces) as 0% for each individual experiment. Statistical differences were calculated using one‐way ANOVA (analysis of variance). Asterisk denotes the fact that the recovery values for a previously described patient mutant (309stop) were calculated from the previously published BRET values^2^ for comparison. (**F**) Representative images showing the subcellular localization of the wild‐type EGFP‐PI4K2A enzyme (top) and the indicated mutant forms (10‐μm scale bars). [Color figure can be viewed at wileyonlinelibrary.com]

Patient 1 is a 4‐month‐old boy of Pakistani origin, born to consanguineous parents (Fig. 1A). He had neonatal‐onset intractable seizures and severe global developmental delay (GDD) without milestones, and died at 4 months from respiratory infection. Orofacial dyskinesia was not reported by the family, possibly due to the early death, the predominance of seizures, or lack of reference for normal developmental comparison. Brain MRI was not performed. However, the MRI of an elder sibling showed subtle abnormal signals involving the bilateral periventricular white matter, with edema and mild suboptimal sulci formation observed in the bilateral frontal regions. Metabolic investigations, including urinary organic acids and plasma amino acids, were normal. Genetic testing revealed a homozygous variant in the *PI4K2A* gene ENST00000370631.3:c.1243C>T/p.Gln415Ter (Table 1). His three male siblings shared identical DEDE presentations and outcomes, all of whom died at 4 months of age due to seizures.

**TABLE 1:** Clinical and genetic features of patients with *PI4K2A* variants

	Patient 1	Patient 2	Alkhater et al., patient II:8	Alkhater et al., patient II:9	Dafsari et al., family 1	Dafsari et al., family 2
*PI4K2A* variant (NM_018425.3), zygosity	c.1243C>T/p.Gln415Ter, hom	c.989_990del/p.Thr330ArgfsTer27, hom	c.65C>A; p.(Ser22Ter), hom	c.65C>A; p.(Ser22Ter), hom	c.925C>T p.(Arg309Ter), hom	c.925C>T p.(Arg309Ter), hom
Consanguinity of parents, ethnic background	Yes, Pakistani	Yes, Saudi Arabian	Yes, Saudi Arabian	Yes, Saudi Arabian	Yes, Iranian	Yes, Lebanese
Sex	Male	Male	Male	Male	Female	Female
Age at last follow‐up	4 mo	28 mo	14 y	9 y	2 y 10 mo	2 y
Height at last follow‐up (cm)	49.5 (−3.9 SD)	67 (−5.1 SD)	N/A	N/A	74 (−5.5 SD)	80 (−2.3 SD)
Weight at last follow‐up (kg)	3.1 kg (−4.8 SD)	5.8 (−5.8 SD)	N/A	N/A	5 (−8.7 SD)	10 (−1.7 SD)
Head circumference at last follow‐up (cm)	37 (−4.3 SD)	43 (−3.1 SD)	N/A	N/A	42 (−6.9 SD)	48 (−1.3 SD)
Development	Severe global DD; no sitting, walking, or language	Severe global DD; no sitting, walking, or language	Motor and speech delay; sits unsupported, no walking	Motor and speech delay; sits unsupported, no walking	Motor and speech delay; no sitting or walking	Motor and speech delay; no sitting or walking
Hypotonia	Hypertonia	Hypertonia	Axial hypotonia	Axial hypotonia	Axial hypotonia	Axial hypotonia
Seizures	Generalized and multifocal intractable sz since day 1	No	Generalized sz since 9 y	Generalized sz since 9 y	Tonic, myoclonic intractable sz since 16 m	Epileptic spasms at 6 mo of age
Seizure treatment	PB and LEV	No treatment	LEV, good response	LEV, good response	Partial response to CLB (1.5 y); initial short‐term responses to LEV, primidone, VGB, PB, TPM	Poor to no response to VGB, TPM, LMT
Movement disorders	No	Frequent orofacial dyskinesia and upper‐limb motor stereotypes	Myoclonus, episodic arm dystonia (improved with CLZ), orofacial dyskinesia	Myoclonus, episodic arm dystonia (improved with CLZ), orofacial dyskinesia	Spasticity, episodic arm dystonia, orofacial dyskinesia	Spasticity, orofacial dyskinesia
Brain MRI	N/A	Microcephaly with hypoplasia of the corpus callosum and pons	Hypoplastic corpus callosum, thickened lamina terminalis, loss of cingulate gyral folding, small pons and brainstem, mega cisterna magna	N/A	Dysgenesis of corpus callosum, hypoplastic commissures, diffuse white matter loss, ventriculomegaly, rotated thalami, brainstem and vermis hypoplasia, mega cisterna magna	Callosal dysgenesis with foreshortened segments, absent cingulate gyri, commissural hypoplasia, septum pellucidum agenesis, white matter loss, brainstem and cerebellar hypoplasia, mega cisterna magna
Perform activities of daily living	No	No	No	No	No	N/A

Abbreviations: SD, standard deviation; hom, homozygous; N/A not available; DD, developmental delay; sz, seizures; PB, phenobarbital; LEV, levetiracetam; CLB, clobazam; VGB, vigabatrin; LMT, lamotrigine; CLZ, clonazepam; TPM, topiramate; MRI, magnetic resonance imaging.

Patient 2 is a 28‐month‐old boy of Saudi descent, born to consanguineous parents (first cousins) with no family history of similar conditions (Fig. 1A). He exhibited severe GDD, persistent orolingual dyskinesia (lip/tongue movements), dystonic upper‐limb extension, and stereotypies (Videos 1 and 2), with microcephaly, corpus callosum thinning, and hypoplastic pons on MRI (Fig. 1B). Additionally, bilateral optic nerve atrophy was observed. Additional findings included moderate hearing loss, visual impairment (unable to fix or follow objects), and feeding difficulties. Particularly, patient 2 has never exhibited seizures. No metabolic abnormalities were detected. Genetic analysis identified a homozygous *PI4K2A*:c.989_990del/p.Thr330ArgfsTer27 variant, with both parents confirmed as heterozygous carriers (Table 1).

**VIDEO 1:** Patient 2. (**A**) While seated, the patient exhibits repetitive, sustained, and involuntary movements, initially characterized by side‐to‐side lip movements followed by tongue protrusion. These movements are continuous and persist even while engaging in other activities with the upper limbs. (**B**) In the supine position, dystonic extension movements of the upper limbs are observed along with persistent orolingual dyskinesias.

**VIDEO 2:** Patient 2. (**A**) In the supine position, the patient exhibits repetitive, sustained, and irregular involuntary movements with a sudden onset, involving the lips and frequent tongue protrusion. These movements are accompanied by side‐to‐side head oscillations and continuous, stereotyped upper‐limb movements, characterized by clenched fists and repetitive hand opening and closing, which may correspond to stereotypies. (**B**) In the second part of the video, these orofacial and hand movements become more prominent while maintaining the previously described characteristics. Particularly, the movements persist even during voluntary actions, such as smiling. (**C**) In a seated position, at another time, both orofacial and upper‐limb movements remain present.

Prior cases (n = 4)^2^, ^3^ consistently exhibited orolingual dyskinesia, severe GDD, and variable seizures/myoclonus (Table 1). All 5 living patients exhibited orolingual dyskinesia, suggesting it as a hallmark feature when survival permits its manifestation.

### Functional Characterization of *PI4K2A* Variants in Endosomal PI4P Regulation

To test the functionality of the mutated *PI4K2A* proteins, we generated GFP‐ and iRFP‐fused versions of the wild‐type and mutant (C1243T and C989,990del) proteins. The mutated proteins were then expressed in *PI4K2A* knockout HEK293‐AT1 cells^17^ and examined using either confocal microscopy or BRET analysis to estimate PI4P levels in Rab7‐positive endosomes. Confocal microscopy showed that the GFP‐tagged wild‐type enzyme expressed in *PI4K2A* knockout cells exhibited the endosomal localization characteristic of the distribution of the proteins.^1^, ^2^ In contrast, the C1243T‐mutated enzyme that results in an early stop codon at Q415 exhibited no association with any membrane compartment (Fig. 1C). Curiously, the c.989,990del, which introduces a frameshift at the R329 residue with the addition of a stretch of 25 amino acids (LGGGEGACYQGGCHRQWAGLPTEASstop) before a stop codon, mostly eliminated membrane association, although a small residual association with endosomes was still visible in cells expressing extremely low level of the protein (Fig. 1F). As shown previously,^17^, ^18^, ^19^ *PI4K2A* generates most of the PI4P in Rab7‐positive endo‐lysosomal compartments. There, we showed that wild‐type *PI4K2A* but not a catalytically inactive mutant was able to restore PI4P levels in the Rab7‐positive endosomes.^17^ For those studies we employed a method to monitor the PI4P content in the Rab7‐positive compartment using BRET analysis. This method is based on the energy transfer between a Rab7‐targeted Venus protein and a Luciferase‐fused PI4P‐recognizing protein binding module isolated from *Legionella* (P4M‐2x),^18^ as described previously.^19^ This approach is based on the use of GSK‐A1, a selective inhibitor of PI4KA, the enzyme that generates the plasma membrane (PM) pool of PI4P. Inhibition of PI4KA with GSK‐A1 causes a decrease in PM PI4P, causing the Luciferase‐fused PI4P binding module to relocalize from the PM to other PI4P‐containing membranes, including the Rab7 compartment, which increases the BRET signal in that compartment. Figure 1D shows that *PI4K2A* K/O cells exhibit a modest increase in the BRET signal monitored in the Rab7 compartment after GSK‐A1 addition (blue traces), which reflects the function of the PI4K2B enzyme.^17^ Expression of the wild‐type iRFP‐fused *PI4K2A* substantially increased the amount of PI4P in the Rab7 compartment (red traces). In contrast, the C1243T mutant enzyme failed to restore the PI4P levels in the Rab7 compartment (purple traces). However, the c.989_990del mutant protein was able to restore the response partially (green traces). The extent of restoration was calculated from these traces using area under the curve measurements for the time periods indicated by the gray box (plotted in Fig. 1E) for statistical analysis. We also included in this graph values obtained from calculations of earlier data that we previously published on another patient mutation (309 stop)^2^ (marked by an asterisk). These results clearly showed that the mutated proteins are either inactive or functionally severely compromised.

### Orolingual Dyskinesia in *PI4K2A* ‐RD and Beyond

Orolingual dyskinesia was universal in living *PI4K2A*‐RD patients, absent only in patient 1 due to early death. Literature review (Tables S1 and S2) revealed its underreporting in DEDEs and NDDs, despite presence in conditions like *GNAO1*‐ and *PDE10A*‐related disorders.

## Discussion

This study expands the understanding of *PI4K2A*‐RD through two new cases, solidifying its classification as a DEDE. All 6 patients exhibited severe developmental delay and variable seizures, with functional assays tying *PI4K2A* variants to disrupted endosomal PI4P regulation—a vital trafficking process.^17^ The complete loss of function of the C1243T variant correlates with the lethal phenotype of patient 1, whereas the partial activity of the C989_990del variant aligns with survival and dyskinesia of patient 2, suggesting genotype–phenotype gradients.

A prominent feature emerging from *PI4K2A*‐RD is orolingual dyskinesia, observed consistently in all living patients. Unlike many DEDEs^6^, ^20^, ^21^, ^22^, ^23^ where such movements are underreported, its presence in *PI4K2A*‐RD offers diagnostic utility, potentially aiding earlier recognition. The pathophysiology remains unclear, but our data link mutant *PI4K2A* functionality to severity: C1243T fails to restore Rab7‐positive endosomal PI4P, whereas C989_990del retains partial activity, possibly acting as a dominant negative by engaging partners like AP‐3 or GABARAP ineffectively. This could amplify vesicular trafficking defects, driving dyskinesias and other symptoms. Beyond trafficking, roles of *PI4K2A* in autophagy and lysosomal repair suggest broader disruptions in neuronal homeostasis, warranting further exploration.

In contrast, orolingual dyskinesias in other DEDEs, such as *GNAO1*‐related disorders, appear as paroxysmal events tied to dyskinetic crises,^11^, ^24^ unlike *PI4K2A*‐RD, which shows a continuous pattern. *FOXG1*‐related conditions share some features, but *PI4K2A*‐RD growth impairment and neuroimaging findings distinguish it.^9^ Nongenetic causes (eg, drug induced, autoimmune) differ contextually from genetic disorders like *PI4K2A*‐RD. Across NDDs, the under‐recognition of orolingual dyskinesia, compounded by inconsistent terminology,^4^ limits its diagnostic potential, highlighting the need for standardized descriptions.

Management of orolingual dyskinesia remains a challenge, as pharmacological treatments such as tetrabenazine, baclofen, or benzodiazepines rarely provide sustained benefits. Nonpharmacological approaches, including nutritional support and speech‐language therapy, offer symptomatic relief but do not address the underlying cause. In our cohort, no specific treatments alleviated orolingual dyskinesias, underscoring the urgent need for innovative, mechanism‐based therapies.

The study, despite expanding the cohort, is limited by its small sample size of 6 patients (2 new and 4 previously reported), which restricts the generalizability of findings and the ability to definitively characterize the full phenotypic spectrum of *PI4K2A*‐RD or accurately determine the prevalence of orolingual dyskinesia. Although the rarity of the condition is acknowledged, this small sample size significantly limits the statistical power of the conclusions.

This study underscores orolingual dyskinesias as a hallmark of *PI4K2A‐RD*, enhancing diagnostic precision and clinical management. Increased awareness and further characterization of these movements in neurodevelopmental research could refine diagnostic approaches and uncover potential therapeutic avenues for these complex disorders.

## Author Roles

(1) Research Project: A. Conceptualization, B. Design, C. Organization, D. Execution, E. Review and critique, (2) Statistical Analysis: A. Design, B. Execution, C. Review and Critique. (3) Manuscript preparation: A. Writing of the first draft, B. Review and critique

R.M.: 1A, 1B, 1C, 1D, 2B.

J.D.O.‐E.: 1E, 2A.

P.R.: 1E, 2B.

J.R.A.: 1E, 2B.

A.M.M.: 1E, 2B.

N.A.M.A.: 1E, 2B.

S.E.: 1E, 2B.

T.S.: 1E, 2A, 2B.

T.B.: 1E, 2A, 2B.

H.H.: 1A, 1B, 1C, 1E, 2B.

## Disclosures

**Financial Disclosures:** The authors declare that there are no additional disclosures to report.

**Full financial disclosures of all authors for the previous 12 months:** The authors declare that there are no additional disclosures to report.

## Supporting information


**Table S1.** Clinical features of disorders with orofacial dyskinesias and treatment response.
**Table S2.** Comparative classification of phenotypic similarity among genetic disorders and PI4K2A‐related disorders.

**Figure S1.** The results of the bibliographic search, categorizing the causes of orofacial dyskinesia into DEDEs (developmental and epileptic dyskinetic encephalopathy); other genetic movement disorders (non‐DEDEs); autoimmune, paraneoplastic, and postinfectious conditions; drug‐induced causes; and other neurological and systemic conditions. These classifications help in understanding the wide range of potential etiologies for this symptom.

## Data Availability

The data that support the findings of this study are available from the corresponding author on reasonable request.
