# Supplementary material for: Recessive Loss of PI4K2A Function Causes a Developmental and Epileptic Dyskinetic Encephalopathy with Prominent Orolingual Dyskinesia
Source: Mov Disord. 2025 Aug 7;40(10):2243–50. doi: 10.1002/mds.30286 (PMC13001697; doi:10.1002/mds.30286)
Supplement: Supplementary file 3 — Table S1. Clinical features of disorders with orofacial dyskinesias and treatment response. Table S2. Comparative classification of phenotypic similarity among genetic disorders and PI4K2A‐related disorders. [file MDS-40-2243-s001.docx]

**Materials and Methods**

**Cell Culture**

HEK293-AT1 cells, which stably express the rat AT_1a_ angiotensin II receptor {Hunyady, 2002 #3584}, or PI4K2A knockout (K/O) cells (Clone #26; HEK293-AT1 background;{Baba, 2019 #7344}) were cultured in Dulbecco’s Modified Eagle Medium (DMEM-high glucose; Gibco, Life Technologies) containing 10% (vol/vol) FBS and supplemented with a 1% solution of penicillin/streptomycin (Gibco, Life Technologies). Cell lines were maintained at 37°C and 5% CO_2_ in a humidified atmosphere and regularly tested for *Mycoplasma* contamination using a commercially-available detection kit (InvivoGen). Additionally, after thawing, cell cultures are treated with plasmocin (InvivoGen) at 500 µg/ml for the initial three passages (6-9 days) and supplemented with 5 µg/ml of the prophylactic for all subsequent passages.

**Reagents**

Stock solutions of all reagents were dissolved in the indicated solvent and stored in small aliquots at -20°C. GSK-A1, a PI4KA-selective inhibitor {Bojjireddy, 2014 #7150}, was prepared at 100 μM in DMSO, while Coelenterazine h (Regis Technologies) was dissolved in 100% ethanol (vol/vol) at 5 mM.

**DNA Constructs**

Plasmids were constructed by standard restriction cloning using enzymes from New England Biolabs, while site-directed mutagenesis was done using the QuikChange II kit (Agilent). Truncations and point mutations were verified using standard Sanger sequencing (Psomagen, USA). EGFP- or iRFP-tagged mutant forms of PI4K2A (PI4K2A-C1243T and PI4K2A-C989,990del) were generated using the previously described fluorescently-tagged wild-type versions of PI4K2A {Dafsari, 2022 #7710}. For generating the mutant enzymes, the following primers were used for PCR amplification: (Forward Primer: 5’-GCTCTCGGGACAGACTGGGTGGTG-3’; Reverse Primer: 5’-CACCACCCAGTCTGTCCCGAGAGCTAGAAC-3’ for PI4K2A 989-990del, and Forward Primer: 5’-TTCGACAGGGGCTAGTTCCATAAGC-3; Reverse Primer: 5’-ATGGAACTAGCCCCTGTCGAAACC-3’ for the C1243T form). The levels of PI4P in Rab7-positive membrane compartments were measured using the bioluminescence resonance energy transfer (BRET)-based sLuc-P4M2x-T2A-mVenus-Rab7 biosensor described previously {Baba, 2019 #7344}. To prevent any interference with the BRET signal, the iRFP-tagged version of the PI4K2A and its mutant forms were used.

**Live-Cell Confocal Microscopy**

HEK293-AT1 cells (3x10^5^ cells/dish) were plated with a final volume of 1.5 mL on 29 mm circular glass-bottom culture dishes (#1.5; Cellvis) pre-coated with 0.01% poly-L-lysine solution (Sigma). Plated cells were allowed to attach overnight prior to transfection with plasmid DNAs (0.1-0.2 μg/well) using Lipofectamine 2000 (2-5 μL/well; Invitrogen) within a small volume of Opti-MEM (200 μL; Invitrogen). Lipofectamine-complexed DNA was incubated together with the cells for 4-6 h before being exchanged for complete culture medium. After 18-20 h of transfection, cells were incubated in 1 mL of modified Krebs-Ringer solution (containing 120 mM NaCl, 4.7 mM KCl, 2 mM CaCl_2_, 0.7 mM MgSO_4_, 10 mM glucose, 10 mM HEPES, and adjusted to pH 7.4) and images were acquired at room temperature using a Zeiss LSM 980 (63x/1.40 N.A. Plan-Apochromat Oil DIC M27 Objective) laser-scanning confocal microscope together with the Blue-ZEN software system (Carl Zeiss Microscopy).

**Bioluminescence Resonance Energy Transfer (BRET) Measurements**

BRET-based measurements of PI4P levels within Rab7-positive membrane compartments of intact cells has been described in detail previously {Baba, 2019 #7344; Dafsari, 2022 #7710}. Briefly, measurements were made at 37°C using a Tristar2 LB 942 Multimode Microplate Reader (Berthold Technologies) with customized emission filters (540/40 nm and 475/20 nm). PI4K2A K/O cells (0.75x10^5^ cells/well) were seeded in a 200 μL total volume to white-bottom 96 well plates pre-coated with 0.01% poly-L-lysine solution (Sigma) and cultured overnight. Cells were then transfected with 0.25 μg of the sLuc-P4M2x-T2A-mVenus-Rab7 BRET biosensor and 0.01 μg of either iRFP-PI4K2A, its mutant forms or the piRFP-C1 empty vector using Lipofectamine 2000 (1 μL/well) within OPTI-MEM (40 μL). Lipofectamine-complexed DNA was incubated together with the cells for 4-6 h before being exchanged for complete culture medium. At between 20-24 h post-transfection, cells were washed once before being incubated for 30 minutes in 50 µl of modified Krebs-Ringer buffer (containing 120 mM NaCl, 4.7 mM KCl, 2 mM CaCl_2_, 0.7 mM MgSO_4_, 10 mM glucose, 10 mM HEPES, and adjusted to pH 7.4) at 37°C in a CO_2_-independent incubator. After the pre-incubation period, the cell-permeable luciferase substrate, coelenterazine h (40 µl, final concentration 5 µM), was added and a 5 min baseline BRET measurement (30 s/cycle) was recorded. The plates were then quickly unloaded for manual addition of GSK-A1 (30 nM) or a DMSO vehicle control, which were prepared in a 10 µl volume of the modified Krebs-Ringer solution. Measurements were carried out in triplicate wells and repeated as three independent experiments. From each well, the BRET ratio was calculated by dividing the 530 nm and 485 nm intensities, which were then normalized to the baseline measurement. For each iRFP-PI4K2A variant or the iRFP-C1 control, the BRET ratios obtained from GSK-A1-treated wells were normalized to an internal DMSO vehicle control.

**Statistical analysis**

For calculation of statistical differences, the areas under the curve (AUC) were calculated for each individual experiments using the BRET ratio values for the time interval between 1200 and 2000 seconds. Percent recovery was then calculated using the wild-type recovery (red traces in **Fig. xB**) as 100% and the recovery with the empty iRFP plasmid (blue traces) as 0%. These percent recovery values from the three separate experiments were than analyzed by one way ANOVA with multiple comparisons (GraphPadPrism v10).

**Literature search**

To investigate whether orofacial dyskinesias are present in other acquired or genetic disorders, a literature search was conducted in PubMed using the following terms: orofacial dyskinesia, orofacial chorea, orobuccolingual dyskinesia, buccolingual dyskinesia, orofacial hyperkinesia, facial dyskinesia, perioral dyskinesia, and lingual-facial-buccal dyskinesia. The search covered articles from 1951 to February 2025 with the filter applied: Child: birth-18 years. Articles were selected based on title and abstract, excluding those with unclear etiology. Additionally, these keywords were used in OMIM to gather more diseases that may not have been found in the PubMed search.

**Supplementary Table 1. Clinical features of disorders with orofacial dyskinesias and treatment response**

| *Disorders (gene, reference), inheritance* | *Age at onset* | *Clinical characteristics* | *Associated Symptoms* | ***Treatment Response*** |
| --- | --- | --- | --- | --- |
| Ceroid lipofuscinosis, neuronal,13 (*CTSF*, (1)), AR | Adult-onset | Transient perioral dyskinesias | Tonic-clonic seizures, cerebellar dysarthria, cognitive decline, dementia, and segmental myoclonic jerks. | No |
| Parkinson disease 15, autosomal recessive (*FBXO7*, (2)), AR | 19 y | Perioral dyskinesias | Difficulty walking (right lower limb dragging), abnormal neck movements, slow and monotonous voice, postural tremors in both upper limbs, abnormal body posturing, asymmetric pyramidal weakness, and cogwheel rigidity in all four limbs. No memory impairment. | L-dopa, trihexyphenidyl, mild improvement |
| *ADCY5*-related disorders (*ADCY5*, (3,4)), AD | Early childhood or adolescence | Facial, perioral, and periorbital; confirmed by EMG in some cases. | Paroxysmal, jerky, choreiform, or dystonic; worsened by anxiety, head tremor, hyperactive reflexes and gait instability | CBZ, VPA, propranolol, primidone, amitriptyline, trifluoperazine, chlordiazepoxide, and acetazolamide. No improvement |
| Neurodevelopmental disorder with impaired speech and hyperkinetic movements (*ZNF142*, (5)), AR | Adult onset | Tremulous cervical dystonia, facial dyskinesia | Intellectual disability, speech impairment, seizures, tremor and dystonia | No |
| Parkinsonism-dystonia, infantile, 2 (*SLC18A2*, (6)), AR | Childhood onset | Facial dyskinesia | Severe parkinsonism and no ambulation, mood disturbance, autonomic instability, and developmental delay, | Pramipexole, dramatic improvement |
| Niemann-pick disease, type C2 (*NPC2*, (7)), AR | Adult onset | Mild facial dyskinesia | Dysarthria, bradykinesia, vertical supranuclear gaze paresis, dysdiadochokinesis, progressive language impairment, perseveration, echolalia, poor cognitive performance, impaired auditory comprehension, limited speech, poor word list generation, impaired visuospatial functions, and visual hallucinations. | No |
| CIMDAG syndrome (*VPS4A*, (8)), AD | Childhood onset | Choreodystonic movements with facial dyskinesia | Global developmental delay, axial hypotonia, peripheral spasticity, microcephaly, no ocular pursuit, early-onset cataracts, retinal dysfunction, cerebral atrophy, thin dysplastic corpus callosum, basal ganglia atrophy, brainstem and cerebellar hypoplasia. | No |
| McLeod syndrome (*XK*, (9)), XL | Adult onset, rarely childhood onset | Facial dyskinesia | Dilated cardiomyopathy, atrial fibrillation, hepatosplenomegaly, muscle weakness, myopathy, chorea, dysarthria, seizures, acanthocytosis, hemolysis, personality disorder, anxiety, depression, OCD. | Tetrabenazine, olanzapine, chlorpromazine, limited efficacy. |
| *GNAO1*-related disorders (10), AD | Childhood onset | Facial dyskinesia, especially during dyskinetic crisis | Global developmental delay, seizures, mixed movement disorders | Benzodiazepines, tetrabenazine, limited efficacy, DBS effective |
| Neurodegeneration with brain iron accumulation 3 *(FTL,* (11)*), AD* | Adult onset, rarely adolescent onset | Buccolingual dyskinesia | Cognitive defects (including frontotemporal/subcortical dementia), parkinsonism, dystonia, cerebellar ataxia, dysarthria, behavioral changes, and dysphagia. | Benzodiazepines, limited efficacy |
| *CACNA2D1-*related disorders, (12)), AR | Childhood onset | ﻿Orofacial dyskinesia | ﻿Hypotonia, microcephaly, absent speech, spasticity, choreiform movements, cortical visual impairment, corpus callosum hypoplasia and progressive volume loss | No |
| Spinocerebellar ataxia 7 (*ATXN7*, (13)), AD | Adult onset | Orofacial dyskinesia | Ataxia, visual failure (with or without ataxia), dysarthria (early onset), brisk deep tendon reflexes, small choreic movements (distal limbs), saccadic slowing progressing to external ophthalmoparesis, progressive visual loss | No |
| Frontotemporal dementia and/or amyotrophic lateral sclerosis 7 (*CHMP2B*, (14)), AD | Adult onset | Orofacial dyskinesia | Behavioral and personality changes, sometimes accompanied by dyscalculia, parkinsonian features such as rigidity, hypokinesia, reduced arm swing, and pyramidal signs | No |
| Dyskinesia, limb and orofacial, infantile-onset (*PDE10A*, (15)), AD/AR | Childhood onset | Orofacial dyskinesia | Delayed motor development, unsteady gait, frequent falls, axial hypotonia, limb and trunk dyskinesias, intermittent chorea and ballismus, intention tremor, drooling, and dysarthria | No |
| Mitochondrial DNA depletion syndrome 17 (*MRM2*, (16)), AR | Childhood onset | Orofacial dyskinesia | Tip-toe walking developmental regression, generalized dystonia, tremors, dysarthria, unintelligible speech, hypotonia, choreiform movements | No |
| Basal ganglia calcification, idiopathic, 1 (*SLC20A2*, (17)), AD | Adult onset | Orolingual dyskinesia | Gait disturbance, parkinsonism, bradykinesia, rigidity, dysarthria, postural instability, micrographia, tremor, mask-like facies, limb dysmetria, chorea, athetosis, extrapyramidal signs, dystonia, pyramidal signs, hyperreflexia, cerebellar ataxia, dysdiadochokinesia, memory impairment, mental deterioration, CT scan dense calcifications in basal ganglia, cerebellar dentate nucleus, thalamus, hippocampus, subcortical white matter, and cortex | No |
| Choreoacanthocytosis (*VPS13A*, (18)), AR | Adult onset | Orofacial dyskinesia | Dysphagia, drooling, pes cavus, limb muscular atrophy and weakness, progressive choreoathetosis, dystonia, parkinsonism, hyporeflexia/ areflexia, dysarthria, seizures, tics, caudate and putamen atrophy, dementia, behavioral and psychiatric manifestations, acanthocytes and increased creatine kinase | Clozapine, quetiapine, tetrabenazine, deutetrabenazine, BZD, limited efficacy. Botulinum toxin |
| *TLE1*-related disorders ((19)), AR | Childhood onset | Orofacial dyskinesia necessitating tube feeding | Postnatal microcephaly, profound developmental delay, hypotonia, failure to thrive, intermittent opisthotonic posturing, peripheral hypertonia, myoclonus (no seizures), progressive cerebral atrophy, delayed myelination, and spastic quadriplegia | No |
| Dystonia 31 (*AOPEP*, (20)), AR | Childhood and adult onset | Orofacial dyskinesias | Facial and neck dystonia, swallowing difficulties, muscle cramping and pain, multifocal or generalized dystonia (affecting upper and lower limbs, trunk), walking difficulties, stiffness, abnormal posturing, dysarthrophonia, speech articulation defects, hypophonia, and late-onset parkinsonism. | Anticholinergics (partial response), tetrabenazine (partial response), botulinum toxin(no response) |
| Myopathy with extrapyramidal signs (*MICU1*, (21)), AR | Childhood onset | Orofacial dyskinesias | Delayed motor development, proximal muscle weakness, learning difficulties, progressive extrapyramidal motor signs (chorea, tremor, dystonic posturing), ataxia, microcephaly, ophthalmoplegia, ptosis, optic atrophy, and axonal peripheral neuropathy. Laboratory findings: Increased serum creatine kinase and myopathic muscle biopsy findings | No |
| Spinocerebellar ataxia 27A (*FGF14*, (22)), AD | Childhood and adult onset | Orofacial dyskinesias | Ataxia, unsteadiness, aggressive outbursts, depression, dysmetric saccades, disrupted ocular pursuit, gaze-evoked nystagmus, cerebellar dysarthria, high-frequency small-amplitude hand tremor, head tremor, cerebellar atrophy on MRI | No |
| Neurodegeneration, early-onset, with choreoathetoid movements and microcytic anemia  (*IREB2*, (23)), AR | Adolescent – young adult onset | Orofacial dyskinesias | Neonatal feeding difficulties, delayed psychomotor development, hypotonia, dystonic posturing, choreoathetoid movements (upper limbs and face), hypertonia, spasticity (lower limbs), tonic-clonic seizures, corticospinal tract signs (hyperreflexia, extensor plantar responses), mild dysmorphic features, progressive cerebral volume loss, delayed myelination, retinal dysfunction, and chronic refractory microcytic hypochromic anemia. | Treatment-resistant choreoathetoid movement disorder |
| *PRRT2*-related disorders, (24), AD/AR | Childhood onset | Paroxysmal dyskinesia, including face | Epilepsy, mixed and paroxysmal movement disorder, and migraine. | CBZ |
| Dystonia 30 (*VPS16*, (25)), AD | Childhood onset | Orofacial dyskinesia | Early-onset multifocal dystonia, torticollis, bulbar dystonia, dysphonia, swallowing difficulties | No |
| Neurodegeneration with brain iron accumulation 1 (*PANK2*, (26)), AR | Childhood onset | Orofacial movements | Spasticity, progressive loss of walking, language deterioration, generalized dystonia, pigmentary retinopathy and the "eye of the tiger" sign on brain MRI, and acanthocytosis | No |
| Basal ganglia calcification, idiopathic, 8, autosomal recessive (*JAM2*, (27)), AR | Adult onset | Orofacial dyskinesia | Anxiety, agitation, headache, dizziness, memory dysfunction, vomiting, urinary incontinence, slurred speech, bradykinesia, hypokinesia. CT: calcifications in the bilateral basal ganglia, thalamus, dentate nuclei, subcortical white matter, and midbrain | No |
| *FOXG1*-related disorders (28), AD | Childhood onset | Orofacial dyskinesia | Microcephaly, mixed movement disorders (stereotypes, chorea, dystonia), intellectual disability, seizure, and corpus callosum dysmorphism | No |
| Neurodegeneration, childhood-onset, stress-induced, with variable ataxia and seizures *(ADPRS,*(29)*), AR* | Childhood onset | ﻿Facial mini-myoclonus | ﻿Dystonia, sudden diplopia, dizziness, ataxia, gait instability, ﻿progressive hearing loss, urinary urgency, thoracic kyphoscoliosis, ﻿muscle weakness and atrophy of hands and feet, leg spasticity with clonus, truncal and appendicular ataxia, and spastic-ataxic gait. | No |
| Rapid-onset dystonia parkinsonism *(ATP1A3,* (30)*), AD* | Childhood and adult onset | Orofacial dystonia | Sudden-onset dystonia with postural instability, severe bulbar symptoms (dysarthria, drooling), hypertonicity, hyperreflexia, onset triggered by stress in some cases, stable progression in most patients | No |
| Developmental delay and seizures with or without movement abnormalities *(DHDDS,* (31)*), AD* | Childhood onset | ﻿Frequent, irregular, single, short duration (<100 ms) polymorphic bursts (myoclonus) | Short stature, hypotonia, global developmental delay, multiple types of seizures, intellectual disability, speech delay, involuntary movements, ataxia, tremor, dystonia, bradykinesia, and rigidity | No |
| *KCNMA1-related disorders* (32)*, AD* | Childhood onset | Facial grimacing | Seizures, ASD, dystonia, paroxysmal attacks | Psychostimulant therapy, CBZ, acetazolamide, caffein, variable response |
| Benign hereditary chorea *(NKX2-1,* (33)*), AD* | Childhood onset | Facial dyskinesia | Hypothyroidism, neonatal respiratory distress syndrome, motor delay, hypotonia | Tetrabenazine, L-dopa, methylphenidate, partial response |
| Niemann-pick disease, type C1 (*NPC1,* (34)*), AR* | Childhood onset | Facial dyskinesia | Neurological regression, hepatomegaly, hypotonia, unexplained falls | No |
| Intellectual developmental disorder, autosomal dominant 55, with seizures *(NUS1,* (31)*), AD* | Childhood onset | Prominent facial myoclonus  involvement, exacerbated  by action + stimulus  sensitive | Delayed development, impaired intellectual development (ranging from borderline to severe), motor delay, language delay, dysarthria, ataxic gait, clumsiness, poor fine motor skills, tremor, myoclonic seizures, and multiple types of seizures, autism spectrum disorder. | No |
| Microcephaly, progressive, seizures, and cerebral and cerebellar atrophy (*QARS1,* (35)*), AR* | Childhood onset | ﻿Orofacial dyskinesia, intermittent mouthing and chewing movements | Microcephaly, seizures, stereotypes | LEV, TPM ineffective |
| Spinocerebellar ataxia 2 *(ATXN2,* (36)*), AD* | Adult onset, rarely young patients | Facial fasciculations | Ataxia, ophthalmoparesis, neuropathy, dystonia, spasticity, dysphagia | No |
| Spinocerebellar ataxia 5 *(SPTBN2,* (37)*), AD* | Adult onset, rarely young patients | Facial myokymia | Hypotonia, ataxia, intention tremor, nystagmus | No |
| Spinocerebellar ataxia 15 *(ITPR1,* (38)*), AD* | Adult onset | ﻿Facial myokymia and ﻿buccolingual dyskinesias | Gait ataxia, ﻿gaze-evoked horizontal nystagmus, hyperreflexia, dysphagia | No |

AD: autosomal dominant, AR: autosomal recessive, ASD: autism spectrum disorders; BZD: benzodiazepine, CBZ: carbamazepine; DBS: deep brain stimulation, LEV: levetiracetam; TPM: topiramate; VPA: valproic acid

**References**

1. Fabio R Di, Moro F, Pestillo L, Meschini MC, Pezzini F, Doccini S, et al. Pseudo-dominant inheritance of a novel CTSF mutation associated with type B KUFS disease. Neurology. 2014;83(19):1769–70.

2. Panagariya A, Sharma B, Dev A. Pallido-pyramidal syndrome : A rare entity. Indian J Med Sci . 2007;61(3):156-7.

3. Fernandez M, Raskind W, Wolff J, Matsushita M, Yuen E, Graf W, et al. Familial dyskinesia and facial myokymia (FDFM): A novel movement disorder. Ann Neurol. 2001;49(4):486–92.

4. Koht J, Løstegaard SO, Wedding I, Vidailhet M, Louha M, Tallaksen CME. Benign hereditary chorea, not only chorea: A family case presentation. Cerebellum and Ataxias. 2016;3(1):1–7.

5. Khan K, Zech M, Morgan AT, Amor DJ, Skorvanek M, Khan TN, Hildebrand MS, Jackson VE, Scerri TS, Coleman M, Rigbye KA, Scheffer IE, Bahlo M, Wagner M, Lam DD, Berutti R, Havránková P, Fečíková A, Strom TM, Han V, Dosekova P, Gdovinova Z, Laccone F, Jameel WJ. Recessive variants in ZNF142 cause a complex neurodevelopmental disorder with intellectual disability, speech impairment, seizures, and dystonia. Genet Med. 2019;21(11):2532–2542.

6. Rilstone JJ, Alkhater RA, Minassian BA. Brain Dopamine–Serotonin Vesicular Transport Disease and Its Treatment. N Engl J Med. 2013;368(6):543–50.

7. Klünemann HH, Elleder M, Kaminski WE, Snow K, Peyser JM, O’Brien JF, et al. Frontal lobe atrophy due to a mutation in the cholesterol binding protein HE1/NPC2. Ann Neurol. 2002;52(6):743–9.

8. Lunati A, Petit A, Lapillonne H, Gameiro C, Saillour V, Garel C, et al. VPS4A mutation in syndromic congenital hemolytic anemia without obvious signs of dyserythropoiesis. Am J Hematol. 2021;96(4):E121–3.

9. Vaisfeld A, Bruno G, Petracca M, Bentivoglio AR, Servidei S, Vita MG, et al. Neuroacanthocytosis Syndromes in an Italian Cohort: Clinical Spectrum, High Genetic Variability and Muscle Involvement. Genes (Basel). 2021;12(3).

10. Domínguez Carral J, Reinhard C, Ebrahimi-Fakhari D, Dorison N, Galosi S, Garone G, et al. Dyskinetic crisis in GNAO1-related disorders: clinical perspectives and management strategies. Front Neurol . 2024;15(June):1–11.

11. Vidal R, Ghetti B, Takao M, Brefel-Courbon C, Uro-Coste E, Glazier BS, et al. Intracellular Ferritin Accumulation in Neural and Extraneural Tissue Characterizes A Neurodegenerative Disease Associated with A Mutation in the Ferritin Light Polypeptide Gene. J Neuropathol Exp Neurol. 2004;63(4):363–80.

12. Mayo S, Gómez-Manjón I, Marco-Hernández AV, Fernández-Martínez FJ, Camacho A, Martínez F. N-Type Ca Channel in Epileptic Syndromes and Epilepsy: A Systematic Review of Its Genetic Variants. Int J Mol Sci. 2023;24(7).

13. Enevoldson TP, Sanders MD HA. Autosomal dominant cerebellar ataxia with pigmentary macular dystrophy. A clinical and genetic study of eight families. Brain. 1994;117(3):445-60.

14. Fujioka S WZ. Clinical aspects of familial forms of frontotemporal dementia associated with parkinsonism. J Mol Neurosci. 2011;45(3):359-65.

15. Diggle CP, Sukoff Rizzo SJ, Popiolek M, Hinttala R, Schülke JP, Kurian MA, et al. Biallelic Mutations in PDE10A Lead to Loss of Striatal PDE10A and a Hyperkinetic Movement Disorder with Onset in Infancy. Am J Hum Genet. 2016;98(4):735–43.

16. Shafique A, Arif B, Chu ML, Moran E, Hussain T, Zamora FM, et al. MRM2 variants in families with complex dystonic syndromes: evidence for phenotypic heterogeneity. J Med Genet. 2023;60(4):352–8.

17. Woo KA, Yoo D, Lee JY, Kim MJ, Seong MW, Park SS, et al. SLC20A2 mutation manifesting as very late-onset orofacial dyskinesia. Neurol Sci. 2021;42(6):2561–4.

18. Kim A, Chae HY, Park HS. Compound Heterozygous VPS13A Variants in a Patient with Neuroacanthocytosis: A Case Report and Review of the Literature. Lab Med. 2022;53(4):433–5.

19. Cavallin M, Maillard C, Hully M, Philbert M, Boddaert N, Reilly ML, et al. TLE1, a key player in neurogenesis, a new candidate gene for autosomal recessive postnatal microcephaly. Eur J Med Genet. 2018;61(12):729–32.

20. Zech M, Kumar KR, Reining S, Reunert J, Tchan M, Riley LG, et al. Biallelic AOPEP Loss-of-Function Variants Cause Progressive Dystonia with Prominent Limb Involvement. Mov Disord. 2022;37(1):137–47.

21. Logan C V., Szabadkai G, Sharpe JA, Parry DA, Torelli S, Childs AM, et al. Loss-of-function mutations in MICU1 cause a brain and muscle disorder linked to primary alterations in mitochondrial calcium signaling. Nat Genet. 2014;46(2):188–93.

22. Van Swieten JC, Brusse E, De Graaf BM, Krieger E, Van de Graaf R, De Koning I, et al. A mutation in the fibroblast growth factor 14 gene is associated with autosomal dominant cerebral ataxia. Am J Hum Genet. 2003;72(1):191–9.

23. Costain G, Ghosh MC, Maio N, Carnevale A, Si YC, Rouault TA, et al. Absence of iron-responsive element-binding protein 2 causes a novel neurodegenerative syndrome. Brain. 2019;142(5):1195–202.

24. Yang K, Quiroz V, Ebrahimi-fakhari D. PRRT2-Related Disorder. 2018 Jan 11 [Updated 2024 Jul 4] Adam MP, Feldman J, Mirzaa GM, al, Ed GeneReviews® [Internet] Seattle Univ Washington, Seattle; 1993-2025.

25. Li LX, Jiang LT, Liu Y, Zhang XL, Pan YG, Pan LZ, et al. Mutation screening of VPS16 gene in patients with isolated dystonia. Park Relat Disord. 2021;83(December 2020):63–5.

26. Higgins JJ, Patterson MC, Papadopoulos NM, Brady RO, Pentchev PG, Barton NW. Hypoprebetalipoproteinemia, acanthocytosis, retinitis pigmentosa, and pallidal degeneration (HARP syndrome). Neurology. 1992;42(1):194–8.

27. Khojasteh M, Soleimani P, Ghasemi A, Taghizadeh P, Rohani M, Alavi A. JAM2 variants can be more common in primary familial brain calcification (PFBC) cases than those appear; may be due to a founder mutation. Neurol Sci. 2024;45(8):3829–44.

28. Spagnoli C, Fusco C, Pisani F. Pediatric-Onset Epilepsy and Developmental Epileptic Encephalopathies Followed by Early-Onset Parkinsonism. Int J Mol Sci. 2023;24(4).

29. Lindskov FO, Karlsson WK, Skovbølling SL, Nielsen EN, Dunø M, Stokholm J, et al. Expanding the Spectrum of Stress-Induced Childhood-Onset Neurodegeneration with Variable Ataxia and Seizures (CONDSIAS). Cerebellum. 2024;23(2):861–71.

30. Pittock SJ, Joyce C, Keane VO, Hugle B, Hardiman O, Brett F, et al. Rapid-onset dystonia-parkinsonism: a clinical and genetic analysis of a new kindred. Neurology. 2000;55(7):991-5.

31. Williams LJ, Waller S, Qiu J, Innes E, Elserafy N, Procopis P, et al. DHDDS and NUS1: A Converging Pathway and Common Phenotype. Mov Disord Clin Pract. 2024;11(1):76–85.

32. Roze E, Silveira-Moriyama L, Leu-Semenescu S, Villeneuve N, Lecardonnel B, François-Heude MC, et al. KCNMA1-Related Episodes of Behavioral Arrest and Loss of Postural Reflexes: A Critical Reappraisal. Mov Disord Clin Pract. 2024;12(November 2024):215–25.

33. Patel NJ, Jankovic J. NKX2-1-Related Disorders. GeneReviews®. University of Washington, Seattle; 2016.

34. Sheth JJ, Sheth FJ ON. Niemann-Pick type C disease. Indian Pediatr. 2008;45(6):505-7.

35. Chan DL, Rudinger-Thirion J, Frugier M, Riley LG, Ho G, Kothur K, et al. A case of QARS1 associated epileptic encephalopathy and review of epilepsy in aminoacyl-tRNA synthetase disorders. Brain Dev. 2022;44(2):142–7.

36. Geschwind DH, Perlman S, Figueroa CP, Treiman LJ, Pulst SM. The prevalence and wide clinical spectrum of the spinocerebellar ataxia type 2 trinucleotide repeat in patients with autosomal dominant cerebellar ataxia. Am J Hum Genet. 1997;60(4):842–50.

37. Jacob FD, Ho ES, Martinez-Ojeda M, Darras BT, Khwaja OS. Case of infantile onset spinocerebellar ataxia type 5. J Child Neurol. 2013;28(10):1292–5.

38. Di Gregorio E, Orsi L, Godani M, Vaula G, Jensen S, Salmon E, et al. Two Italian families with ITPR1 gene deletion presenting a broader phenotype of sca15. Cerebellum. 2010;9(1):115–23.

**Supplementary Table 2.** Comparative classification of phenotypic similarity among genetic disorders and *PI4K2A*-related disorders

| Classification | Criteria | Genes |
| --- | --- | --- |
| Highly similar phenotypes | Disorders presenting with nearly identical clinical features, including movement disorders (orolingual dyskinesia) and shared neurological manifestations. | *CACNA2D1, FOXG1, GNAO1, MRM2, QARS1, TLE1, VPS4A,* |
| Moderately similar Phenotypes | Disorders with overlapping yet distinguishable phenotypic features, including variations in movement abnormalities, severity, or associated comorbidities. | *ADCY5, ADPRS, AOPEP, DHDDS, FGF14, MICU1, NUS1, PDE10A, SLC18A2*, |
| Minimally similar or not similar | Disorders that exhibit distinct phenotypic differences, limited overlapping clinical features, or significantly divergent neurological manifestations. | *ATP1A3, ATXN2, ATXN7, CHMP2B, CTSF, FBXO7, FTL, IREB2, ITPR1, KCNMA1, NKX2-1, NPC1, NPC2, PANK2, PRRT2, SLC20A2*, *SPTBN2, VPS13A, VPS16*, *XK, ZNF142* |
