# Supplementary figures and images for: Recessive Loss of PI4K2A Function Causes a Developmental and Epileptic Dyskinetic Encephalopathy with Prominent Orolingual Dyskinesia
Source: Mov Disord. 2025 Aug 7;40(10):2243–50. doi: 10.1002/mds.30286 (PMC13001697; doi:10.1002/mds.30286)

# Orofacial dyskinesia

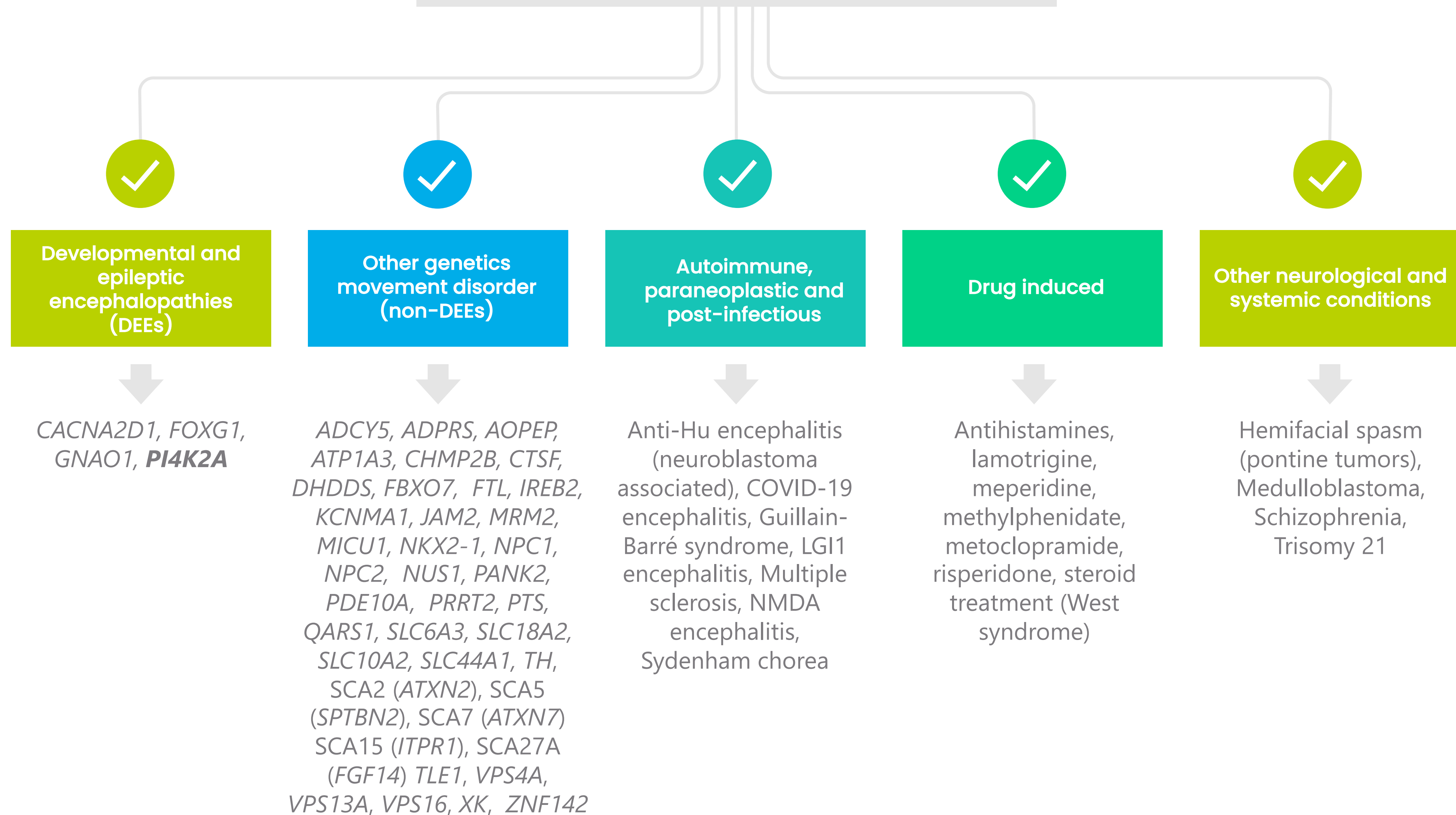

Supplement: Supplementary file 4 — Figure S1. The results of the bibliographic search, categorizing the causes of orofacial dyskinesia into DEDEs (developmental and epileptic dyskinetic encephalopathy); other genetic movement disorders (non‐DEDEs); autoimmune, paraneoplastic, and postinfectious conditions; drug‐induced causes; and other neurological and systemic conditions. These classifications help in understanding the wide range of potential etiologies for this symptom. [file MDS-40-2243-s002.pdf]
